# Sapovaccarin-S1 and -S2, Two Type I RIP Isoforms from the Seeds of *Saponaria vaccaria* L.

**DOI:** 10.3390/toxins14070449

**Published:** 2022-06-30

**Authors:** Louisa Schlaak, Christoph Weise, Benno Kuropka, Alexander Weng

**Affiliations:** 1 Institute of Pharmacy, Freie Universität Berlin, Königin-Luise-Str. 2+4, 14195 Berlin, Germany; l.schlaak@fu-berlin.de; 2 Institute of Chemistry and Biochemistry, Freie Universität Berlin, Thielallee 63, 14195 Berlin, Germany; chris.weise@biochemie.fu-berlin.de (C.W.); kuropka@zedat.fu-berlin.de (B.K.)

**Keywords:** plant toxin, ribosome-inactivating protein (RIP), type I RIP, rRNA glycosylase activity (EC 3.2.2.22), protein isolation, protein sequencing, mass spectrometry

## Abstract

Type I ribosome-inactivating proteins (RIPs) are plant toxins that inhibit protein synthesis by exerting rRNA *N*-glycosylase activity (EC 3.2.2.22). Due to the lack of a cell-binding domain, type I RIPs are not target cell-specific. However once linked to antibodies, so called immunotoxins, they are promising candidates for targeted anti-cancer therapy. In this study, sapovaccarin-S1 and -S2, two newly identified type I RIP isoforms differing in only one amino acid, were isolated from the seeds of *Saponaria vaccaria* L. Sapovaccarin-S1 and -S2 were purified using ammonium sulfate precipitation and subsequent cation exchange chromatography. The determined molecular masses of 28,763 Da and 28,793 Da are in the mass range typical for type I RIPs and the identified amino acid sequences are homologous to known type I RIPs such as dianthin 30 and saporin-S6 (79% sequence identity each). Sapovaccarin-S1 and -S2 showed adenine-releasing activity and induced cell death in Huh-7 cells. In comparison to other type I RIPs, sapovaccarin-S1 and -S2 exhibited a higher thermostability as shown by nano-differential scanning calorimetry. These results suggest that sapovaccarin-S1 and -S2 would be optimal candidates for targeted anti-cancer therapy.

## 1. Introduction

*Saponaria vaccaria* L., also known as cow cockle or prairie carnation, is an annual herbaceous plant belonging to the carnation family (Caryophyllaceae). The flowering plant is a single species in its genus *Vaccaria* and was originally widespread in Eurasia, but nowadays is also found in North and South America, South Africa, and Australia [1]. Many different synonyms for *Saponaria vaccaria* L. exist in the literature, for example *Vaccaria pyramidata* Medik., *Gypsophila vaccaria* (L.) Sm, and *Vaccaria hispanica* (Mill.) Rauschert. For more than 1000 years, Wang-Bu-Liu-Xing (*Vaccariae semen* in Chinese) have been used in traditional Chinese medicine to treat dysmenorrhea, amenorrhea, and lactation failure [1]. The seeds of *Saponaria vaccaria* L. contain triterpenoid saponins including gypsogenin bisdesmosides, cyclic peptides, flavonoids, and polysaccharides [1,2,3,4]. In 1995, Bolognesi et al. reported on a 28 kDa type I ribosome-inactivating protein (RIP) from the seeds of *Vaccaria pyramidata* Medik. for the first time [5].

RIPs are distributed in over more than 100 species [6]. However, they have been discovered primarily in the families of Caryophyllaceae, Cucurbitaceae, Euphorbiaceae, Fabaceae, Phytolaccaceae, and Poaceae [6]. RIPs exhibit rRNA *N*-glycosylase activity (EC 3.2.2.22). It was first shown on rat ribosomes that RIPs cleave off a specific adenine residue (A_4324_) from a conserved GAGA motif in the alpha-sarcin loop of the 28S rRNA [7]. As a consequence, the integrity of the 60S ribosomal subunit is compromised, the elongation factor 2 cannot interact with the ribosome any longer and the translation process at the ribosome is stalled [8]. As a result of the cleavage protein synthesis is inhibited, which in the end leads to cell death [8].

A distinction is made between type I and type II RIPs. Type II RIPs such as ricin from *Ricinus communis* L. are composed of an enzymatically active A-chain and a B-chain with lectin-like properties which promotes the binding to galactose molecules on the cell surface [9,10]. That allows them to enter the cell by receptor-mediated endocytosis, using the retrograde transport to the endoplasmic reticulum and reach the cytosol by the endoplasmic-reticulum-associated protein degradation (ERAD) pathway to exert their cytotoxicity [11,12]. Type I RIPs are enzymatically active single-chain proteins [13]. Lacking the B-chain, they enter the cell by receptor-independent endocytosis and accumulate in the late endosomes and lysosomes, where degradation takes place [14]. Only a small quantity escapes from the lysosomes which results in low cytotoxicity [14]. Very prominent and well-characterized type I RIPs are dianthin 30 from *Dianthus caryophyllus* L. and saporin-S6 from *Saponaria officinalis* L. [15,16].

The primary function of RIPs in the plant is not yet completely clear [17]. Different hypotheses on the role of RIPs in plants are under discussion. RIPs possess antiviral, antifungal, antibacterial and insecticidal activities, which may contribute in the protection against plant pests and predators [18,19]. In addition, RIP activity is increased in senescent and stressed leaves, suggesting that RIPs play an important role in regulating the death of plant cells. Also, roles as regulators of protein synthesis and protein storage have been proposed [20].

RIPs have increasingly become a focus of research due to their very efficient protein synthesis inhibitory activity and the resulting high cytotoxicity. Currently, research is being conducted primarily with regard to their promising application in targeted anti-tumor therapy as conjugates with target-specific antibodies, so-called immunotoxins. More than 450 immunotoxins constructed with RIPs are published in the literature [21]. Most of the described immunotoxins have been tested in vitro, some of them also in vivo. Only a few RIP-immunotoxins have been used for clinical studies [21]. Because of the high relevance of this topic, there is a great interest in the scientific community to explore and characterize new RIPs with the aim of identifying additional suitable candidates for immunotoxins. Beyond their potential in anti-cancer therapy, a second area of application for plant extracts containing saponins and RIPs arose in the last few years: the use of RIPs for crop protection. The new application was derived from the function that RIPs have in the plants, in which they are synthesized. Antiviral properties of RIPs have been demonstrated for a wide range of plant viruses [22]. The RIP plant extract can either be directly applied to the plant surface or, alternatively, a virus-resistant transgenic plant may be constructed [23,24]. In terms of crop protection, the antiviral activity of RIPs is complemented by insecticidal effects [19,25].

In 1995, the group of Fiorenzo Stirpe succeeded in isolating a type I RIP from *Saponaria vaccaria* L. (*Vaccaria pyramidata* Medik.) with a size of approximately 28 kDa and an isoelectric point (pI) of 9.5 and could identify its 30 N-terminal amino acids [5]. In 1997, the isolated RIP was used for immunoconjugate construction and successful inhibition of tumor growth in mice by these immunotoxins was demonstrated [26]. The full amino acid sequence, however, has never been published. The authors of the same study reported increased cytotoxicity of the isolated RIP from *Saponaria vaccaria* L. when compared to other RIPs on different cell lines [5]. Due to the increased cytotoxicity, RIPs from *Saponaria vaccaria* L. appear to be promising candidates for use in targeted tumor therapy and crop protection. We now aimed to isolate the type I RIP from *Saponaria vaccaria* L., to determine its precise molecular mass and its complete amino acid sequence, and to characterize its thermal stability and enzymatic activity. Additionally, we attempted to investigate the distribution of type I RIPs across differently processed seed material, as well as identify the main location of type I RIPs within the seed. The main location of type I RIPs within the seed has never been characterized before.

## 2. Results

### 2.1. Protein Extraction from the Seeds of Saponaria vaccaria L.

**Figure 1:**
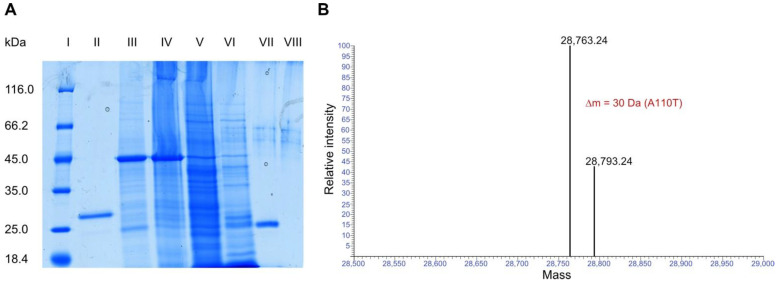
Isolation of sapovaccarin-S1 and -S2 from the seeds of *Saponaria vaccaria* L. and determination of its protein mass. (**A**) SDS-PAGE (12.5%) of the protein purification of sapovaccarin-S1 and -S2 using the whole-seed fraction, Coomassie Brilliant Blue stain. I: Protein marker (in kDa); II: N-terminally His-tagged dianthin (His-dianthin; 0.66 µg), a type I RIP from *Dianthus caryophyllus* L., as a reference; III: Crude extract from *Saponaria vaccaria* L. (4.83 µg); IV: 30%-ammonium sulfate fraction (40.03 µg); V: 60%-ammonium sulfate fraction (45.76 µg); VI: 90%-ammonium sulfate fraction (28.06 µg); VII: The 90%-ammonium sulfate fraction was subjected to cation exchange chromatography. At 0.3 M NaCl sapovaccarin-S1 and -S2 eluted (0.55 µg); VIII: Blank, phosphate buffered saline (PBS). (**B**) High-resolution mass spectrometric analysis of the isolated protein revealed two mono-isotopic masses of 28,763.24 Da and 28,793.24 Da. The y-axis represents the relative signal intensity and the x-axis shows the deconvoluted mass in Da.

Whole dried seeds of *Saponaria vaccaria* L. were available for protein purification. A crude extract with complex protein composition was obtained by aqueous extraction. In SDS-PAGE the crude extract showed promising protein bands in the mass range of 20–35 kDa typical for RIPs (Figure 1A, lane III) [27]. In order to further separate and concentrate these proteins, the aqueous extraction was followed by an ammonium sulfate precipitation at three different concentrations (30%, 60% and 90%). Similar to the crude extract, the three ammonium sulfate fractions also revealed protein bands in the expected mass range for RIPs (Figure 1A, lane IV–VI). The 90%-ammonium sulfate fraction displayed the strongest bands in the mass range of interest (Figure 1A, lane VI). Similar results were obtained in qualitative analysis of the *N*-glycosylase activity. While all three fractions exhibited *N*-glycosylase activity, the 90%-ammonium sulfate fractions possessed by far the highest enzyme activities and therefore were chosen for further protein isolation [28]. These were further subjected to cation exchange chromatography (see Supplementary Figures S1 and S2). At a concentration of 0.3 M NaCl (in 50 mM HEPES, pH 7.0), an apparent single protein eluted with a molecular mass of approximately 25 kDa as estimated from SDS-PAGE (Figure 1A, lane VII). This band had already been among the most intense bands in the crude extract as well as in the 90%-ammonium sulfate fraction. The final yield of protein isolated from the 90% fraction was 8.56 mg per 100 g of whole seeds. We were also able to demonstrate enzyme activity for the isolated protein fraction. High-resolution mass spectrometry of the isolated protein yielded a mono-isotopic mass of 28,763.24 Da and an additional mass peak of 43% relative abundance at 28,793.24 Da (mass difference Δm = 30 Da, Figure 1B), suggesting that the isolated protein fraction contains two RIPs that in the following will be referred to as sapovaccarin-S1 and -S2.

### 2.2. Protein Sequencing of Sapovaccarin-S1 and -S2

**Figure 2:**
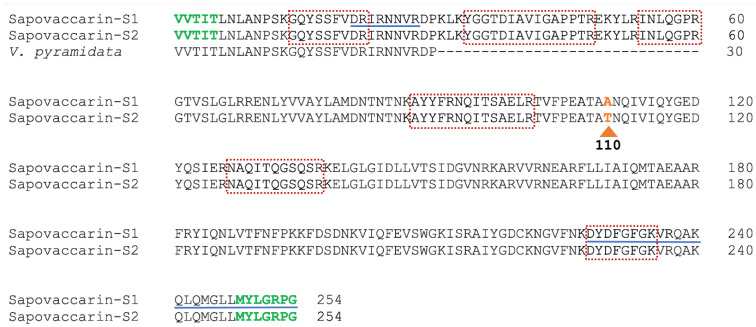
Protein sequence determination of sapovaccarin-S1 and -S2. Sequence alignment of sapovaccarin-S1 and -S2 and the N-terminus of *Vaccaria pyramidata* Medik. identified by Bolognesi et al. [5]. The final sequences of sapovaccarin-S1 and -S2 are the result of combining MS-based peptide analysis and PCR experiments. Amino acids shown in black were identified by PCR experiments and amino acids (N-terminal and C-terminal region) highlighted in green were not covered by PCR analysis. Peptide sequences identified by MALDI-MS-based peptide sequencing are highlighted by red boxes. Amino acids which could be confirmed by ISD data are underlined in blue. In addition, a triangle is highlighting the amino acid at position 110, indicating the position where sapovaccarin-S1 (alanine) and -S2 (threonine) differ.

At the beginning of this study, only the 30 N-terminal amino acids sequenced by Bolognesi et al. had been identified [5]. Here, the determination of the complete amino acid sequence of sapovaccarin-S1 and -S2 was achieved by combining MS-based peptide analysis with PCR experiments. The isolated protein was in-gel digested with trypsin and the peptide map recorded by MALDI-TOF-MS. The resulting peptide mass fingerprint was compared to a protein sequence database using the software Mascot. Since some peptide masses could be matched with known type I RIP sequences, it was confirmed that the isolated protein is a type I RIP. However, many dominant peptide signals from the spectra could not be assigned to any known RIP sequence indicating that the isolated type I RIP has a different sequence. Additionally, the sequences of six tryptic peptides with 64 amino acid residues were determined de novo by MS/MS analysis. Based on the identified peptide sequences, two PCR experiments were performed (see Supplementary Figure S3). DNA of gypsophilin-S, a type I RIP from *Gypsophila elegans* M.Bieb., saporin-S6 and dianthin 30 served as templates for primer design [29,30,31]. Both experiments combined resulted in a 728-bp DNA sequence that was translated into the 242 amino acid sequence shown in Figure 2.

This sequence was named sapovaccarin-S1. In the N- and C-terminal region, five and seven amino acids, respectively, could not be identified by the described PCR method (Figure 2). MALDI in-source decay (ISD) was conducted in order to obtain additional information on the N- and C-terminal regions. C-terminal ISD measurements revealed an ion series, enabling to complete the C-terminus (see Supplementary Figure S4). The N-terminus published by Bolognesi et al. matched the sequence identified by PCR and ISD data, finally allowed to confirm the five missing N-terminal residues (Figure 2). The complete protein sequence of mature sapovaccarin-S1 consisting of 254 amino acids is shown in Figure 2.

In addition, in-gel digestions by LysC, chymotrypsin and AspN were performed and peptides were analyzed by LC-ESI-MS. Combining these data sets with the trypsin digest and allowing for unspecific cleavage in the database search, we obtained a sequence coverage of 100% for the sapovaccarin-S1 sequence, thus confirming its correctness. In the trypsin digest, for the peptide with a mass of 2842.4 (pos. 102–126, TVFPEATA**A**NQIVIQYGEDYQSIER) we consistently found a second form with lower signal intensity (roughly one third) with a mass of 2872.4 (∆m = 30 Da) (see Supplementary Figure S5). In the fragment spectrum of this peptide, the b ions beyond b8 and the y ions beyond y16 were shifted by exactly 30 Da, indicative of a substitution A > T at position 110 (see Supplementary Figures S6 and S7). The protein carrying this substitution is referred to as sapovaccarin-S2 (Figure 2). The sapovaccarin-S2 isoform could be detected not only at the peptide level, but also at the DNA level: the codon for A110 revealed two peaks at position 328—a major guanine and a minor adenine peak (see Supplementary Figure S8). The substitution of GCG (sapovaccarin-S1) to ACG (sapovaccarin-S2) implies the amino acid substitution from alanine to threonine at position 110.

As shown in Figure 1B, Orbitrap-based intact protein measurement yielded a mono-isotopic mass of 28,763.24 Da, which is almost identical to the theoretical mass calculated from the obtained sapovaccarin-S1 sequence (28,763.12 Da). Additionally, a minor peak was observed (43% relative abundance compared with the main peak) at a monoisotopic mass of 28,793.24 Da which represents sapovaccarin-S2.

**Figure 3:**
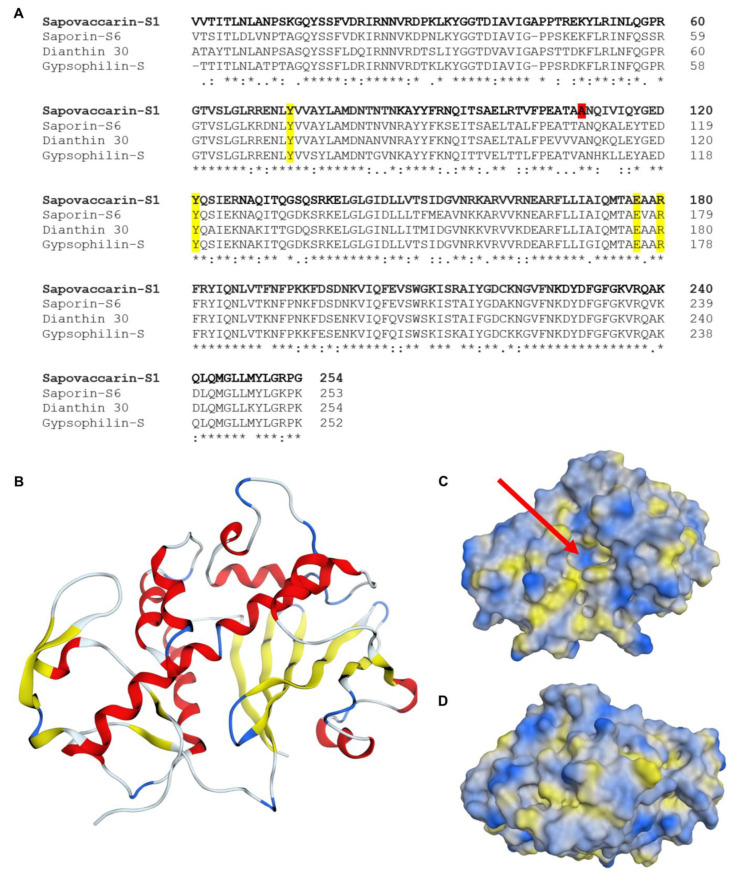
Sequence comparison of sapovaccarin-S1 with well-characterized type I RIPs; tertiary structure model of sapovaccarin-S1. (**A**) Sequence alignment of sapovaccarin-S1, saporin-S6, dianthin 30 and gypsophilin-S. Highly conserved active-site resiudes are highlighted in yellow [33]. The alanine at position 110, substitued in sapovaccarin-S2, is highlighted in red. Aligned amino acids labeled with a star symbol (*) are fully conserved in all four sequences; those with a dot symbol (.) are identical in three out of four sequences and those with colon symbol (:) show moderate identity. Alignment was performed using the Clustal Omega multiple sequence alignment tool (https://www.ebi.ac.uk/Tools/msa/clustalo/, accessed on 11 January 2022). (**B**) Homology model of sapovaccarin-S1 showing its tertiary structure. (**C**) Front view on the protein surface of sapovaccarin-S1. Hydrophilic regions of the protein are highlighted in blue and lipophilic areas in yellow. The substrate binding pocket (red arrow) is located in the cavity in the middle of the protein. (**D**) Back view on the protein surface.The panels (**B**–**D**) have been produced using MOE (version 2020.0901, Chemical Computing Group, Montreal, Canada).

The sequence of sapovaccarin-S1 determined here is highly similar to other well-characterized type I RIP sequences with 83% sequence identity to gypsophilin-S and 79% sequence identity to each saporin-S6 and dianthin 30, as shown in Figure 3A [29,31,32]. The protein sequence data reported in this paper will appear in the UniProt Knowledgebase under the accession numbers Q7M1L6 and C0HM39.

To predict the protein structure of sapovaccarin-S1 a homology model was built (Figure 3B). The high-resolution crystal structure of dianthin 30 (1.4 Å, PDB ID 1RL0) was chosen as template [33]. The template sequence showed 79% identity and 95% similarity with the target sequence of sapovaccarin-S1—an excellent starting point for homology modelling. The root-mean-square deviation (rmsd) between the template structure and the homology model was 0.52 Å. The low rmsd value indicated a correct global fold of the final model. The protein geometry did not show any Phi-Psi outliers nor atom clashes. The three-dimensional structure of sapovaccarin-S1 corresponded to the common fold of type I RIPs consisting of two domains: the N-terminal domain rich in β-strands and the C-terminal domain rich in α-helices (Figure 3B). The active site was located at the cleft between both domains (Figure 3C). In type I and II RIPs the active-site key residues Tyr73, Tyr121, Glu177 and Arg180 are highly conserved (Figure 3A) [33]. The same applied to sapovaccarin-S1—all key residues were preserved at the same position. The protein surface of sapovaccarin-S1 with its hydrophilic binding pocket is shown in Figure 3C,D. The same structural elements were known from dianthin 30 and saporin-S6 (PDB ID 1QI7) [33,34].

### 2.3. N-Glycosylase Activity

**Figure 4:**
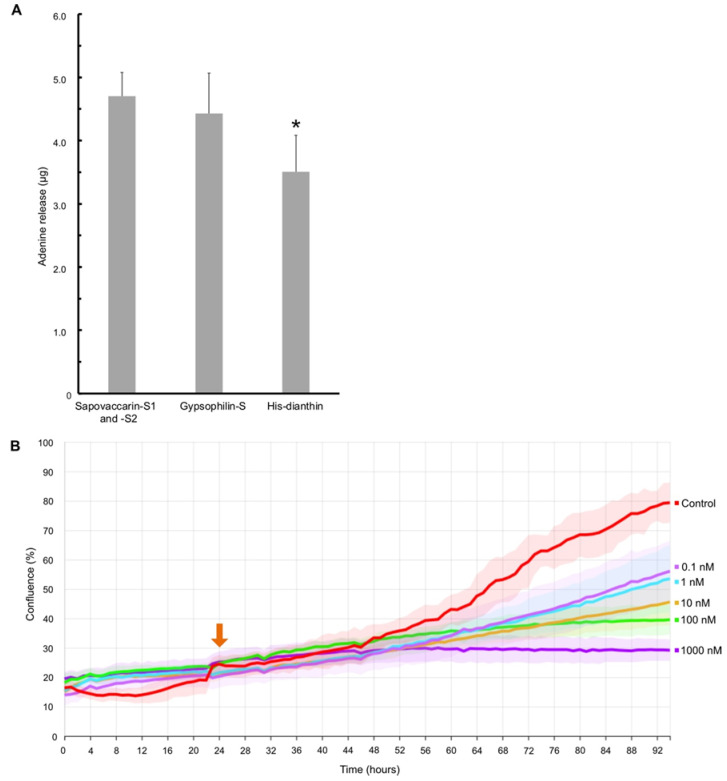
Characterization of the enzymatic activity of sapovaccarin-S1 and -S2. (**A**) Quantitative analysis of *N*-glycosylase activity on an A30-oligonucleotide substrate by TLC-densitometry. Sapovaccarin-S1 and -S2 exhibited *N*-glycosylase activity. Gypsophilin-S and His-dianthin were used as positive controls. Sapovaccarin-S1 and -S2 and gypsophilin-S (each 0.01 nM), that were isolated from the seeds, exhibited significantly higher enzymatic activity than His-dianthin (0.01 nM) that was recombinatly expressed. * significant to sapovaccarin-S1 and -S2 and to gypsophilin-S, t-test, *p* ≤ 0.05. (**B**) Live-cell imaging of Huh-7 cells. After an incubation of 24 h (orange arrow) sapovaccarin-S1 and -S2 was added at different concentrations (0.1–1000 nM). Cells were continuously monitored for 94 h. Sapovaccarin-S1 and -S2 exhibited a concentration-dependent effect on the cell viability of Huh-7 cells.

Due to their cytotoxic effects type I and type II RIPs have now been studied for over 40 years for their promising use in anti-cancer therapy [35]. The cytotoxic effects of the plant toxins are dependent, on the one hand, on the extent of the endosomal release and on the other hand on their characteristic adenine releasing activity from DNA, RNA, and other polynucleotides [14,36,37]. The group of Fiorenzo Stirpe reported *N*-glycosylase activity on rabbit-reticulocyte lysate and purified rat liver ribosomes for the RIP isolated from *V. pyramidata* [5]. The *N*-glycosylase activity of isolated RIPs from *S. vaccaria* can also be extended to an A30-oligonucleotide substrate (A30). Weng developed an assay, which allows to quantify the enzyme activity of RIPs by measuring the adenine release from an A30-oligonucleotide substrate and the subsequent detection at 260 nm on a thin-layer chromatography (TLC) plate [28]. *N*-glycosylase activity of the isolated sapovaccarin-S1 and -S2 and—for comparative purposes—the isolated gypsophilin-S and recombinant His-dianthin were analyzed with the adenine-releasing assay (Figure 4A). The adenine release of sapovaccarin-S1 and -S2 was higher that of the other investigated RIPs. However, His-dianthin which was recombinantly expressed, released significantly less adenine than the RIPs isolated from the seeds (Figure 4A).

### 2.4. Cytotoxicity of Sapovaccarin-S1 and -S2

In order to confirm the previously proven in vitro *N*-glycosylase activity of sapovaccarin-S1 and -S2 in cells, we used label-free live-cell microscopy to investigate the cytotoxicity of sapovaccarin-S1 and -S2 in Huh-7 cells. The confluence analysis of the raw data was performed by the analysis algorithms of the software package CytoSMART. No effect on cell viability could be observed for the different concentrations shown in Figure 4B 24 h after the addition of sapovaccarin-S1 and -S2. After 35 h, 1000 nM sapovaccarin-S1 and -S2 incubation the confluence began to be reduced significantly compared to the control (*t*-test, *p* ≤ 0.05). For 100 nM, 10 nM, and 1 nM the significant reduction in confluence occurred after 46 h, 45 h, and 47 h, respectively (*t*-test, *p* ≤ 0.05). At the end of the sapovaccarin-S1 and -S2 incubation time, a concentration-dependent cytotoxicity of sapovaccarin-S1 and -S2 was observable. A concentration of 1000 nM had the strongest cytotoxic effect compared to the others (*t*-test, *p* ≤ 0.05).

### 2.5. Thermal Stability

**Figure 5:**
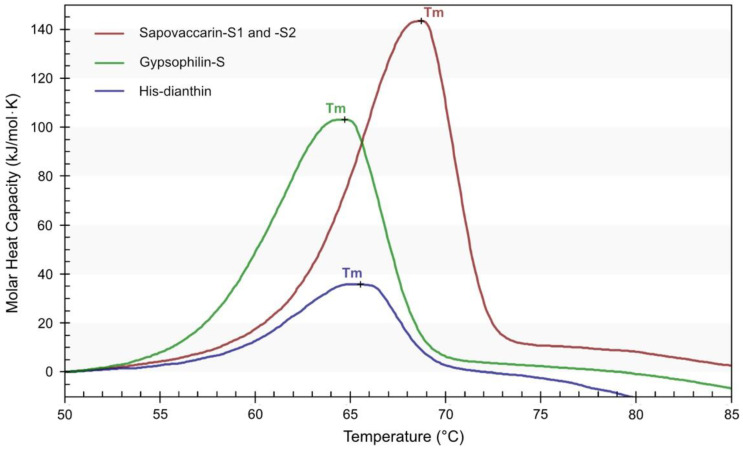
DSC profiles of isolated sapovaccarin-S1 and -S2, isolated gypsophilin-S and recombinantly expressed His-dianthin in PBS, pH 7.4. Each DSC profile was recorded at a protein concentration of 0.4 mg/mL. The transition midpoint temperatures (Tm) were recorded for sapovaccarin-S1 and -S2 at 68.9 °C, for gypsophilin-S at 64.7 °C and for His-dianthin at 65.6 °C. The figure has been produced using NanoAnalyze software (version 3.11.0, TA instruments, New Castle, DE, USA).

Protein thermal stability plays a key role in the development of new anti-cancer drugs, for both science and pharmaceutical industrial processes. The thermostability of sapovaccarin-S1 and -S2 was analyzed by nano-differential scanning calorimetry (DSC). In addition, DSC profiles of gypsophilin-S, which has been isolated recently by our group, and recombinantly expressed His-dianthin were recorded as a reference. The DSC profiles and the transition midpoint temperatures (Tm) are presented in Figure 5. Compared to gypsophilin-S (Tm 64.7 °C) and His-dianthin (Tm 65.6 °C), sapovaccarin-S1 and -S2 had the highest Tm of 68.9 °C. Tm values for proteins range typically between 40 and 80 °C. Thus, all three RIPs possessed a moderately high thermal stability.

### 2.6. Distribution of Sapovaccarin-S1 and -S2 in Differently Processed Seed Material from Saponaria vaccaria L.

**Figure 6:**
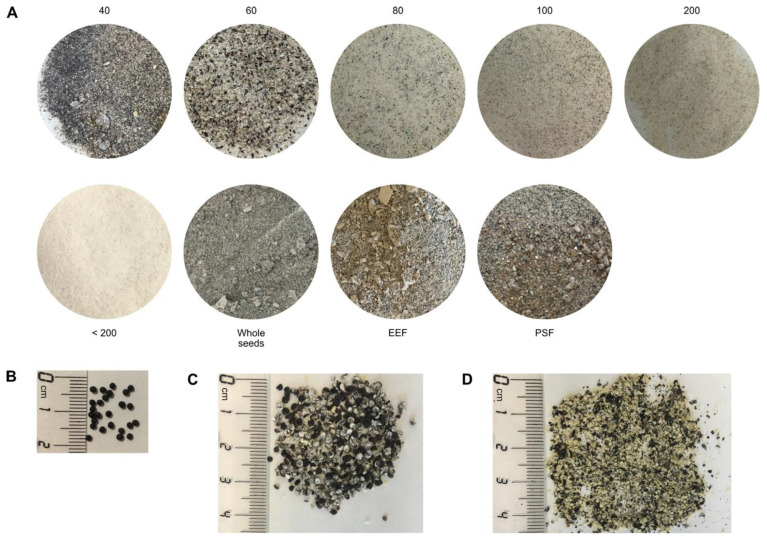
Differently processed seed material from *Saponaria vaccaria* L. (**A**) Defatted and grinded seed material. Fractionated seed material was obtained by sieve analysis: the numbers indicate the mesh sizes of the residues of the fractions or if <200 the passage. The embryo-enriched (EEF) and the perisperm-enriched seed fractions (PSF) were obtained by separating the embryo from the rest of the seed using an impact and a roller mill and by sieving. (**B**) Whole seeds in corresponding scale. (**C**) PSF in corresponding scale. (**D**) EEF in corresponding scale.

Beside the whole seeds, the Canadian Carnation Biocompany provided differently processed seed material, which allowed us to study the exact distribution of sapovaccarin-S1 and -S2 in the processed seed material (see Figure 6). The three ammonium sulfate fractions (30%, 60%, and 90% saturation) of each seed fraction were tested for *N*-glycosylase activity. The whole seeds and all eight seed fractions exerted *N*-glycosylase activity for all three ammonium sulfate fractions. With increasing ammonium sulfate concentration, enzyme activity increased—the highest enzyme activities were consistently found in the 90%-ammonium sulfate fractions. The next aim was to quantify the adenine release activity of the differently processed seed material. For this purpose, the 90%-ammonium sulfate fractions of the whole seeds, the fractionated seed material, the embryo-enriched and the perisperm-enriched seed fraction were investigated. The adenine release correlated with the total protein amount. According to the results shown in Table 1, sapovaccarin-S1 and -S2 was most abundant in the perisperm of the seeds of *Saponaria vaccaria* L.

## 3. Discussion

Here we report the isolation and characterization of sapovaccarin-S1 and -S2, two protein isoforms from *Saponaria vaccaria* L. They were classified as type I RIPs in the course of this study, including their full amino acid sequence. Furthermore, we report for the first time that RIPs in *Saponaria vaccaria* L. are mainly located in the perisperm of the seeds. It should be noted here that the localization of RIPs was determined by evaluating the *N*-glycosylase activity of extracts from perisperm- and endosperm-enriched seed fractions. The localization in the perisperm might also indicate that sapovaccarin-S1 and -S2 could serve for nitrogen storage in *Saponaria vaccaria* L. This observation might lend some support to the hypothesis that RIPs in addition to having a defense function may also function as storage proteins in some plants. Both RIPs were isolated from the seeds by aqueous extraction, ammonium sulfate precipitation, and cation exchange chromatography. The exact amino acid sequence as well the molecular masses of sapovaccarin-S1 and -S2 were determined by PCR and mass spectrometry. Both isoforms differ only in the substitution of one amino acid at position 110 (Ala110 in sapovaccarin-S1 substituted by Thr110 in sapovaccarin-S2). Their intact masses lie within the characteristic mass range of type I RIPs [27]. The ratio of abundance of sapovaccarin-S1 to sapovaccarin-S2 was consistent in the MS spectrum of intact protein mass, the MS/MS spectrum of tryptic peptides and the DNA chromatogram, indicating that sapovaccarin-S1 is the more abundant isoform.

The occurrence of RIP isoforms within the carnation family was already well described in the literature [15,16,38]. The isoforms described herein differ in only one amino acid: the non-polar alanine in position 110 in sapovaccarin-S1 is substituted by a polar threonine residue in sapovaccarin-S2 provoking a minimal increase in mass (∆m = 30 Da) but no changes in pI (calculated pI 9.87). Hence, separation of both isoforms could be achieved neither by cation exchange chromatography nor SDS-PAGE, resulting in a mixture of the two isoforms. Due to the difference in just one amino acid and the resulting very small mass and non-existent pI differences, the separation of both isoforms by other protein purification methods, such as gel filtration or hydrophobic interaction chromatography, probably could not be achieved. We therefore decided to further investigate the mixture of two isoforms. The decision was reinforced by the fact that the single amino acid substitution was located neither within the active site nor in its immediate vicinity. Although in light of the predicted structures it seems highly unlikely that the substitution A110T will have a major impact on *N*-glycosylase activity, in the future this issue might be clarified by producing the individual isoforms recombinantly by site-directed mutagenesis and determining their activity; however, this would fall outside the scope of the present study.

Type I RIPs are mostly encoded by intron-less genes [39]. Therefore, genomic DNA was used as a PCR template. Even though the N- and C-terminal regions were not determined by PCR sequencing, it can be concluded from the PCR data that sapovaccarin-S1 and -S2 are also encoded by intron-less genes. The isoforms described here could be identified from intact protein mass, tryptic peptides as well as from the chromatogram of the PCR analysis. Given that the amino acid substitution at position 110 can be found in the genomic DNA, the isoforms could not have been created by alternative splicing, but had to have been encoded by two different genes.

RIPs from *Saponaria vaccaria* L. were first mentioned by Bolognesi et al. in 1995 [5]. The authors isolated one RIP by aqueous extraction and cation exchange chromatography. Its molecular mass of 28 kDa was determined by SDS-PAGE and gel filtration. In their study, they determined a pI of >9.5 and demonstrated its *N*-glycosidase activity. These results are essentially in agreement with ours. With the applied isolation and mass determination methods, Bolognesi et al. were not able to differentiate the two isoforms. Their assumption that the isolated protein fraction was composed of one protein, underlines the importance of accurate mass spectrometry methods for future research.

Thermostability of sapovaccarin-S1 and -S2 in comparison to gypsophilin-S and His-dianthin was studied by DSC. The Tm of sapovaccarin-S1 and -S2, gypsophilin-S and His-dianthin were determined as 69 °C, 65 °C and 66 °C, respectively. Thus, the isolated mixture of sapovaccarin-S1 and -S2 showed the highest thermal stability among proteins studied. Little data on the thermostability of RIPs has been published. Saporin-S6 and gelonin, a type I RIP from *Gelonium multiflorum*, were analyzed by infrared spectroscopy and two-dimensional correlation spectroscopy at neutral pH (50 mM sodium phosphate buffer, pH 7.4) in two different studies [40,41]. Tm of saporin-S6 was measured at 58 °C and gelonin’s at 66 °C, indicating a moderately high thermostability [40,41]. These data are in accordance with the DSC data of our study. The moderately high thermostability as well as the high *N*-glycosylase activity demonstrated in this study show, that sapovaccarin-S1 and -S2 seem to exhibit potential for toxin moieties in immunotoxins.

## 4. Materials and Methods

### 4.1. Seed Material

The Canadian Carnation BioProducts Company, Saskatoon, S7H 3R2, Canada provided the seeds of *Saponaria vaccaria* L. (Caryophyllaceae). In addition to whole dried seeds, eight differently processed seed fractions were available: six sieve analysis fractions, an embryo-enriched fraction (EEF) and a perisperm-enriched fraction (PSF). The six sieve analysis fractions were obtained by collecting the particles which were stopped by the sieves (mesh size 40, 60, 80, 100 and 200) and the particles which have passed mesh size 200 (<200). The embryo-enriched fraction (EEF) and the perisperm-enriched fractions (PSF), which is the starch and hull fraction of the seed which remains after the embryo is removed, are achieved by separating the embryo from the rest of the seed using an impact and a roller mill and sieving.

### 4.2. Isolation of Sapovaccarin-S1 and -S2

The whole seeds, sieving fraction 40, EEF and PSF had to be ground using an electric mill (M20—Universalmühle, IKA, Staufen, Germany). The raw material of the remaining seed fractions was fine enough from the beginning. Each seed fraction was defatted twice with n-hexane (10 mL/g seed) at 4 °C for 30 min and dried at room temperature. The defatted seed fractions were extracted in PBS pH 7.4 (8 mL/g seed) by gentle stirring at 4 °C over 24 h and thereafter centrifuged at 8000× *g* for 20 min. The resulting supernatant is referred to as ‘crude extract’ and was purified by the following steps: Proteins were separated by fractionated ammonium sulfate precipitation at saturations of 30%, 60% and 90% ammonium sulfate. The precipitated proteins were resuspended in PBS and analyzed by SDS-PAGE (12.5%). The protein fractions were also tested for their *N*-glycosylase activity by measuring the released adenine by TLC densitometry at 260 nm [28]. The 90%-ammonium sulfate fractions showed the highest enzyme activity and were used for further purification by cation exchange chromatography using a prepacked SP Sepharose High Performance column (HiTrap SP XL 1 mL, GE Healthcare Europe, Freiburg, Germany) connected to an ÄKTA start protein purification system (GE Healthcare Europe, Freibug, Germany). Then, 7.0 mL of samples equilibrated with 50 mM HEPES, pH 7.0 were applied to the column and bound proteins were eluted from the column by 0.1 M, 0.2 M, 0.3 M, 0.4 M and 1 M NaCl (in 50 mM HEPES, pH 7.0) at a flow rate of 1 mL/min and detected at 280 nm.

### 4.3. Recombinant Expression of His-Dianthin and Isolation of Gypsophilin-S

N-terminally His-tagged dianthin 30 (His-dianthin) from the plant *Dianthus caryophyllus* L. was recombinantly expressed in E. coli NiCo21(DE3) (New England Biolabs, Ipswich, QLD, USA), purified by Ni-nitrilotriacetic acid affinity chromatography and analyzed by SDS-PAGE as described elsewhere [42]. Gypsophilin-S was isolated from the seeds of *Gypsophila elegans* M.Bieb. using ammonium sulfate precipitation and subsequent ion exchange chromatography. The isolation is described in detail elsewhere [29].

### 4.4. SDS-PAGE and Protein Quantification

12.5% SDS-polyacrylamide gels were used for SDS-PAGE using the Lämmli method. Protein bands were stained with Coomassie Brillant Blue G250 as described elsewhere [43]. Protein concentrations were determined by using a modified Bradford method (ROTI^®^Nanoquant, Carl Roth GmbH, Karlsruhe, Germany).

### 4.5. Protein Mass Spectrometry

Protein and peptide sequences were analyzed by matrix-assisted laser desorption time-of-flight mass spectrometry (MALDI-TOF-MS). All MALDI-TOF-MS measurements were performed with an Ultraflex-II TOF/TOF instrument (Bruker Daltonics, Bremen, Germany) equipped with a 200 Hz solid-state Smart beam^TM^ laser. Data were analyzed using the software FlexAnalysis provided with the instrument. Samples were applied by the dried-droplet technique. Peptides were generated by trypsin, LysC, chymotrypsin and AspN in-gel digestion following a protocol described elsewhere [44]. The mass fingerprints of the generated peptides were recorded in positive reflector mode (RP_PepMix) over an *m/z* range of 600–4000. α-cyano-4-hydroxycinnamic acid was used as matrix. Selected tryptic peptides got analyzed by tandem MS using the LIFT mode [45]. N-terminal c ions and C-terminal (z + 2) ions were generated from the intact and acetone precipitated protein using in-source decay (ISD). As a matrix, 1,5-diaminonaphthalene (1,5-DAN) was used. Spectra were recorded in positive reflector mode (RP_PepMix) over an m/z range of 600–6000.

For high resolution intact protein mass analysis by liquid chromatography-electrospray ionization mass spectrometry (LC-ESI-MS) the isolated protein sample was analyzed using the Ultimate 3000 liquid chromatography system connected to a Q Exactive HF mass spectrometer via the ion max source with HESI-II probe (Thermo Scientific, Waltham, MA, USA). The following MS source parameters were used: spray voltage 3.6 kV, capillary temperature 320 °C, sheath gas 10, auxiliary gas 4, S-lens RF level 60, intact protein mode on. For the analysis 7 µL of a 10 µM protein solution were desalted and concentrated by injection on a reversed-phase cartridge (MSPac DS-10, 2.1 mm × 10 mm, Thermo Scientific, Waltham, MA, USA) at 60 °C using buffer A (0.1% formic acid, 5% acetonitrile in water) at a constant flow rate of 22 µL/min for 3 min. This was followed by a short linear gradient of 5–95% buffer B (0.1% formic acid in 80% acetonitrile, 20% water) within 10 min followed by washing and re-equilibration. Full MS spectra were acquired using the following parameters: mass range m/z 600–2500, resolution 120,000, AGC target 3 × 106, µscans 5, maximum injection time 200 ms. MS raw data were analyzed using BioPharma Finder (version 3.2, Thermo Scientific, Waltham, MA, USA). First, an averaged spectrum over the chromatographic peak was generated followed by spectral deconvolution using a relative abundance threshold of 20% and the function ‘consider overlaps’ turned off.

### 4.6. DNA Extraction from the Seeds and Determination of the DNA Sequence by PCR

Peptide mass fingerprinting and subsequent MS/MS-analysis enabled the identification of first sections of the amino acid sequence of sapovaccarin-S1 and -S2. In order to complete the identified peptides to a full sequence, two PCRs were conducted. Primer pair A was designed based on the peptide mass fingerprint results and primer pair B was derived from the results of the first PCR round and the N-terminus determined by Bolognesi et al. (Table 2). The template DNA was extracted from 75 mg whole dried seeds which were frozen overnight, using the PureLink Plant Total DNA Purification kit (Life Technologies, Carlsbad, CA, USA). DNA concentrations were determined with the NanoDrop (Thermo Fisher Scientific, Waltham, MA, USA). The PCR was conducted using the Phusion High-Fidelity DNA Polymerase (New England Biolabs, Ipswich, QLD, USA). According to the instructions, 25 µL reactions using 64.8 ng of DNA template were prepared. The PCR followed a 3-step protocol with the following cycling steps: Initial denaturation 98 °C for 30 s, denaturation 98 °C for 30 s, annealing for 30 s (annealing temperature see Table 2), extension 72 °C for 22.8 s, final extension 72 °C for 10 min. Denaturation, annealing and extension steps were repeated for 35 cycles. PCR products were separated in a 1% agarose gel. The Monarch Genomic DNA purification Kit (New England Biolabs, Ipswich, QLD, USA) was used to extract the PCR products from the gel. For sequencing purposes extracted PCR product concentrations were determined. PCR products were prepared with corresponding primers and sent to LGC Genomics, Berlin, Germany for sequencing. The Expasy translation tool (https://web.expasy.org/translate/, accessed on 17 September 2021) was used to translate the DNA sequence to a protein sequence.

### 4.7. Homology Modeling

Homology modeling was performed with MOE (version 2020.0901, Chemical Computing Group, Montreal, QC, Canada). The high resolution (1.4 Å) crystal structure of dianthin 30 with PDB ID 1RL0 served as a template). The target sequence and dianthin 30 exhibit 79% sequence identity and 95% sequence similarity. The target sequence and the sequence of dianthin 30 were aligned and checked for correct alignment.

### 4.8. Adenine-Releasing Assay

The *N*-glycosylase activity of different samples was determined by using the adenine-releasing assay as described elsewhere [28]. The assay is based on the cleavage of an adenine from an artificial substrate, a DNA oligonucleotide 5′-A30-3′ (A30). 10 µL of protein sample was mixed with 10 µg A30 (Metabion International AG, Planegg/Steinkirchen, Germany) and filled up to 50 µL with *N*-glycosylase buffer (50 mM sodium acetate, 100 mM KCl, pH 5). Deviating from the publication the mixtures were incubated at 37 °C over night. Samples (each 10 µL) were applied to a TLC 0.25 mm pre-coated silica gel 60 glass plate with fluorescent indicator UV254 (Macherey-Nagel, Düren, Germany) and developed by acetonitrile/water/ammonia (32%), (18:1.6:0.6). In addition, for quantification purposes different adenine amounts (0.125 µg, 0.25 µg, 0.5 µg and 1.0 µg) were applied on the plate. Released adenine was determined by TLC densitometry at 260 nm using the TLCs canner 4 (CAMAG, Berlin, Germany).

### 4.9. Cytotoxicity

To monitor the cytotoxicity of sapovaccarin-S1 and -S2 a label-free live-cell imaging system—the CytoSMART Omni system (CytoSMART Technologies B.V., Eindhoven, Netherlands) was used. The CytoSMART Omni system is an automated brightfield microscope, scanning the complete well surface that can be placed in the incubator. The cytotoxicity studies were performed with Huh-7 cells, a hepatocyte carcinoma cell line. The Huh-7 cell line was obtained from Dr. Mirko Pinotti, University of Ferrara. Then, 8000 cells/well were seeded in 96-well plates, each well containing 150 µL Minimum Essential Medium supplemented with 10% FBS, the plate was placed on the CytoSMART Omni device. Cells were cultured at 37 °C and 5% carbon dioxide. After 24 h sapovaccarin-S1 and -S2 were added (in 20 µL PBS each 3 wells) ranging from 0.1 to 1000 nM (final concentrations). Control cells were only treated with PBS. Image analysis and confluence calculation was performed using the CytoSMART image analysis software package.

### 4.10. Differential Scanning Calorimetry

The thermal stability of protein samples was investigated by differential scanning calorimetry (DSC). A NanoDSC (TA Instruments, New Castle, DE, USA) with capillary cells of 0.3 mL volume was used to carry out the calorimetric measurements. Successive heating and cooling buffer-buffer scans using PBS were repeated three times at a scanning rate of 1 °C/min and over a temperature range of 10–100 °C. During the measurements a total pressure of 3.0 atm was applied to the reference and the sample cell. Protein samples were prepared in PBS with a concentration of 0.4 mg/mL. Prior to measurements buffers and samples were degassed under vacuum for 15 min. A heating scan of each sample was recorded under the same conditions as the buffer-buffer scans. DSC data was analyzed by using the NanoAnalyze software (version 3.11.0, TA instruments New Castle, DE, USA). Buffer-buffer scans got subtracted from each sample scan.

### 4.11. Accession Numbers

The protein sequence data reported in this paper will appear in the UniProt Knowledgebase under the accession numbers Q7M1L6 and C0HM39.

## Figures and Tables

**Table 1:** Adenine released by the 90%-ammonium sulfate fractions of the differently processed seed material.

Seed Fraction	Adenine Release (µg/mg Total Protein)
Whole seeds	720
EEF	99
PSF	851
Mesh size < 200	487
Mesh size 200	639
Mesh size 100	614
Mesh size 80	556
Mesh size 60	285
Mesh size 40	381

**Table 2:** Primer pairs used for sequencing of sapovaccarin-S1 and -S2.

Primer Pairs		Primer Sequence	Annealing Temperature (°C)
A	Forward	5′-AAT GCT AAG ATT ACA CAA GGG-3′	59
	Reverse	5′-GCC CAA ATA CAT AAG GAG TCC C-3′	
B	Forward	5′-CAT TAA ATC TCG CAA ATC C-3′	55
	Reverse	5′-GAC TCC ATC AAT TGA CGT TAC-3′	

## Data Availability

Protein sequencing data have been deposited in UniProt Knowledgebase under the accession numbers Q7M1L6 and C0HM39.

## Supplementary Materials

The following supporting information can be downloaded at: https://www.mdpi.com/article/10.3390/toxins14070449/s1. Figure S1: Cation exchange chromatogram of the isolation of sapovaccarin-S1 and -S2 from the 90% ammonium sulfate fraction of perisperm-enriched seed fraction (PSF). The Y-axis on the left side shows the absorbance at 280 nm in mAU. The Y-axis on the right side represents the composition of the elution buffer in %. 50 mM HEPES, pH 7.0 was used as starting buffer. The NaCl concentration in the elution buffer was gradually increased by adding 1 M NaCl in 50 mM HEPES, pH 7.0. The retention time in min is shown on the X-axis. Sapovaccarin-S1 and -S2 eluted at 0.3 M NaCl. Collected fractions are labeled with 1 to 5; Figure S2: SDS-PAGE (12.5%) of the cation exchange chromatography fractions of sapovaccarin-S1 and S2 using the PSF, Coomassie Brilliant Blue stain. I: Protein marker (in kDa); II: Fraction 1—flow through (6.3–17.5 min); III: Fraction 2 (32.0–35.0 min); IV: Fraction 3 (40.0–42.5 min); V: Fraction 4 (42.5–44.0 min); VI: Fraction 5—sapovaccarin-S1 and -S2 (44.0–46.9 min); VII: Sapovaccarin-S1 and -S2 fraction, concentrated 3 times; VIII: His-dianthin (0.66 µg); Figure S3: DNA sequence of sapovaccarin-S1 obtained by PCR analysis. Based on the tandem MS results, a pair of oligonucleotide primers was designed with the forward primer derived from the DNA sequence of gypsophilin-S (forward primer A) and the reverse primer derived from the C-terminal DNA region of saporin-S6 (reverse primer A). Using these primers and template DNA isolated directly from the seeds of *S. vaccaria* a PCR was performed that yielded a 351-bp PCR product which however did not cover the complete sequence. Therefore, a second primer pair was designed with the reverse primer based on the DNA of the first PCR round (reverse primer B) and the forward primer derived from the DNA of the N-terminus of dianthin 30 (forward primer B). The 449-bp PCR product overlapped in 72 bp with the first PCR product. Combining both sequences resulted in a 728-bp DNA sequence that was translated into a 242-amino acid sequence; Figure S4: In-source decay (ISD) analysis of sapovaccarin (type I RIP from *Saponaria vaccaria* L.). C-terminal ions (z + 2) according to the sequence given in the insert (z9 to z27) and additionally c22 to c28 according to the N-terminal sequence published by Bolognesi et al. are detected; Figure S5: Section of the MS1 spectrum of a tryptic digest of sapovaccarin highlighting the tryptic peptides pos. 102–126 of the isoforms S1 and S2; Figure S6: MS/MS spectra of the tryptic peptide pos. 102–126 of the sapovaccarin isoforms S1 (top) and S2 (bottom). Spectra were analyzed using the Mascot MS/MS search software and the corresponding peptide sequences and the matched b- and y-ions are indicated; Figure S7: Theoretical b- and y-ion series of the tryptic peptide pos. 102–126 of the sapovaccarin isoforms S1 (left) and S2 (right). B-ions differ by 30 Da starting from b9 and y-ions starting from y17; Figure S8: Sequence chromatogram of nucleotides encoding for amino acid positions 107 to 113. At nucleotide position 328 the guanine peak overlays an adenine peak. The codon change from GCG (sapovaccarin-S1) to ACG (sapovaccarin-S2) implicates an amino acid substitution at position 110 from alanine to threonine. Consistent with the results of the trypsin digest and the Orbitrap-based intact protein measurement, the guanine peak is more intense than the adenine peak.
