# Supplementary material for: Sapovaccarin-S1 and -S2, Two Type I RIP Isoforms from the Seeds of Saponaria vaccaria L
Source: Toxins (Basel). 2022 Jun 30;14(7):449. doi: 10.3390/toxins14070449 (PMC9324600; doi:10.3390/toxins14070449)
Supplement: Supplementary file 1 [file toxins-14-00449-s001.zip › toxins-1784256-supplementary.pdf]

toxins

MDPI

Supplementary Information

Sapovaccarin-S1 and -S2, Two Type I RIP Isoforms from the Seeds of *Saponaria vaccaria* L.

Louisa Schlaak, Christoph Weise, Benno Kuropka and Alexander Weng

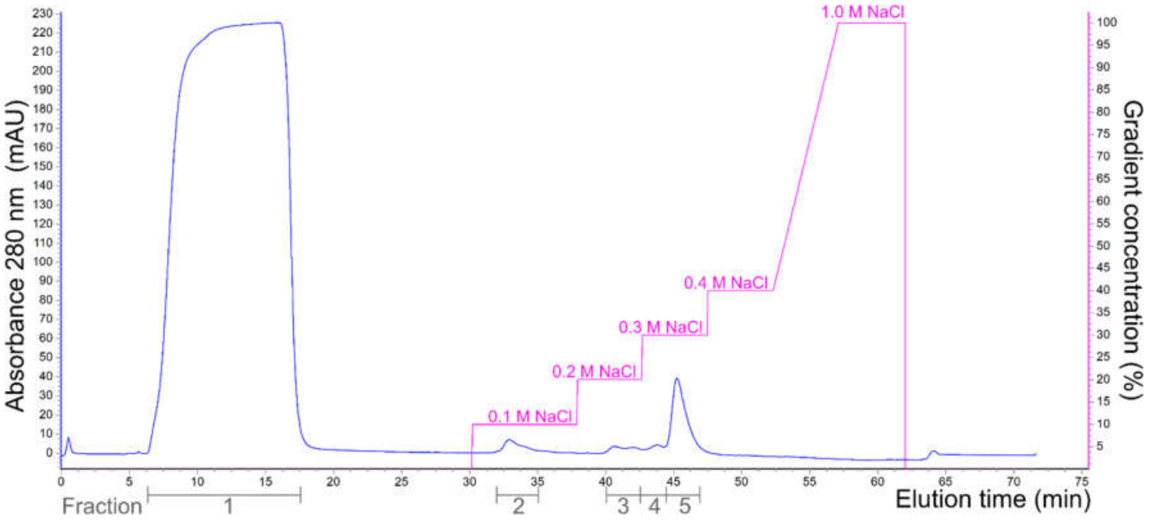

Figure S1. Cation exchange chromatogram of the isolation of sapovaccarin-S1 and -S2 from the 90 % ammonium sulfate fraction of perisperm-enriched seed fraction (PSF). The Y-axis on the left side shows the absorbance at 280 nm in mAU. The Y-axis on the right side represents the composition of the elution buffer in %. 50 mM HEPES, pH 7.0 was used as starting buffer. The NaCl concentration in the elution buffer was gradually increased by adding 1 M NaCl in 50 mM HEPES, pH 7.0. The retention time in min is shown on the X-axis. Sapovaccarin-S1 and -S2 eluted at 0.3 M NaCl. Collected fractions are labeled with 1 to 5.

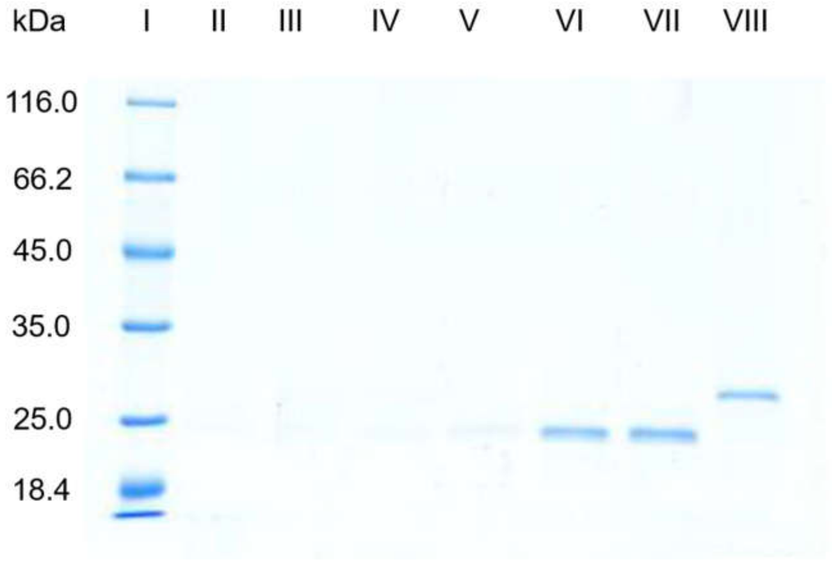

Figure S2. SDS-PAGE (12.5 %) of the cation exchange chromatography fractions of sapovaccarin-S1 and S2 using the PSF, Coomassie Brilliant Blue stain. I: Protein marker (in kDa); II: Fraction 1 - flow through (6.3–17.5 min); III: Fraction 2 (32.0–35.0 min); IV: Fraction 3 (40.0–42.5 min); V: Fraction 4 (42.5–44.0 min); VI: Fraction 5 - sapovaccarin-S1 and -S2 (44.0–46.9 min); VII: Sapovaccarin-S1 and -S2 fraction, concentrated 3 times; VIII: His-dianthin (0.66 μg).

ATTAAATCTCGCAAATCCGAGCAAGGGTCAGTACTCGTCTTTTGTGGATAGAATCCGAAA 60
Forward primer B

CAATGTAAGGGATCCGAAACTGAAATACGGTGGGACTGATATAGCCGTGATAGGGGCGCC 120

GCCTACTCGAGAAAAATACCTTAGAATAAATTTGCAAGGTCCTAGAGGAACAGTCTCACT 180

TGGGCTGAGACGAGAGAATTTGTACGTAGTCGCGTATTTGGCAATGGATAACACGAATAC 240

TAATAAGGCATATTACTTTAGAAATCAAATTACTAGTGCCGAGTTAAGGACCGTTTTCCC 300

CGAGGCCACAGCCGCGAATCAGATAGTTATACAGTACGGGGAAGATTATCAGTCGATAGA 360

AAGGAATGCCCAGATTACACAAGGGAGTCAAAGTAGAAAAGAACTCGGGTTGGGGATCGA 420
Forward primer A

TTTACTTGTAACGTCAATTGATGGAGTCAACAGGAAGGCACGTGTGGTTAGAAACGAAGC 480
Reverse primer B

TAGGTTTCTACTTATCGCTATTCAAATGACGGCTGAGGCAGCGCGTTTTAGGTACATACA 540

GAATTTGGTGACCTTCAACTTTCCTAAGAAGTTCGACTCTGATAACAAGGTGATTCAGTT 600

TGAAGTTAGCTGGGGAAAGATTTCTAGGGCGATATATGGGGATTGCAAAAACGGCGTGTT 660

TAATAAAGATTATGATTTCGGGTTTGGGAAAGTGAGGCAGGCGAAGCAACTCCAAATGGG 720

ACTCCTT 727
Reverse primer A

Figure S3. DNA sequence of sapovaccarin-S1 obtained by PCR analysis. Based on the tandem MS results, a pair of oligonucleotide primers was designed with the forward primer derived from the DNA sequence of gypsophilin-S (forward primer A) and the reverse primer derived from the C-terminal DNA region of saporin-S6 (reverse primer A). Using these primers and template DNA isolated directly from the seeds of *S.vaccaria* a PCR was performed that yielded a 351-bp PCR product which however did not cover the complete sequence. Therefore, a second primer pair was designed with the reverse primer based on the DNA of the first PCR round (reverse primer B) and the forward primer derived from the DNA of the N-terminus of dianthin 30 (forward primer B). The 449-bp PCR product overlapped in 72 bp with the first PCR product. Combining both sequences resulted in a 728-bp DNA sequence that was translated into a 242-amino acid sequence.

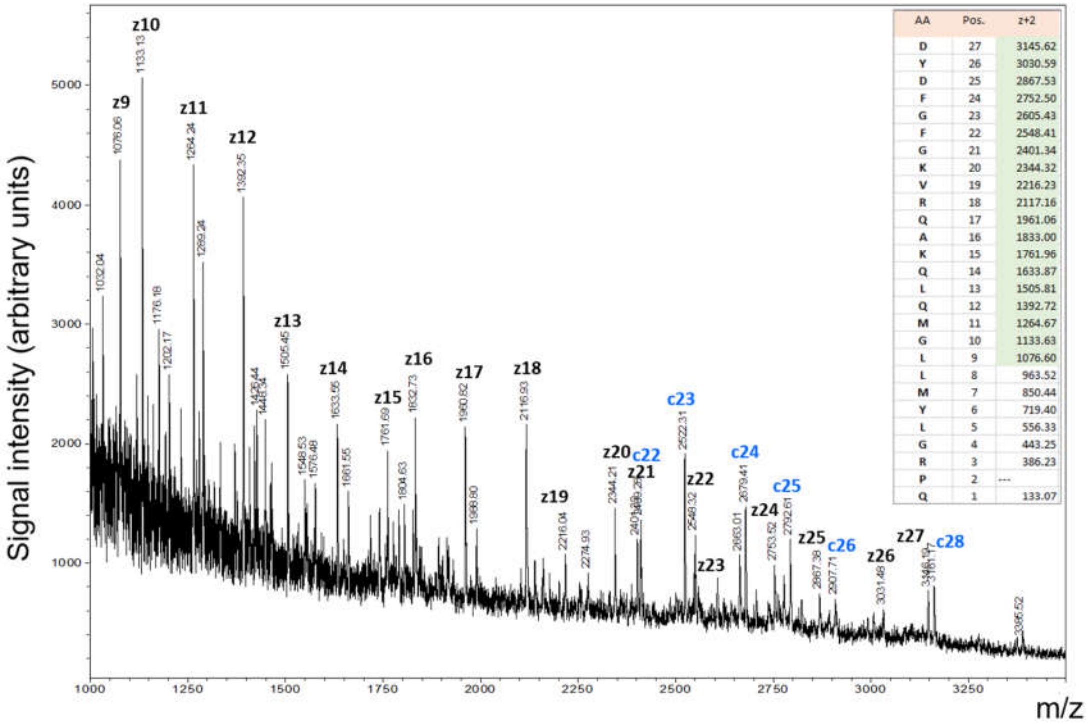

Figure S4. In-source decay (ISD) analysis of sapovaccarin (type I RIP from *Saponaria vaccaria* L.). C-terminal ions (z+2) according to the sequence given in the insert (z9 to z27) and additionally c22 to c28 according to the N-terminal sequence published by Bolognesi *et al.* are detected.

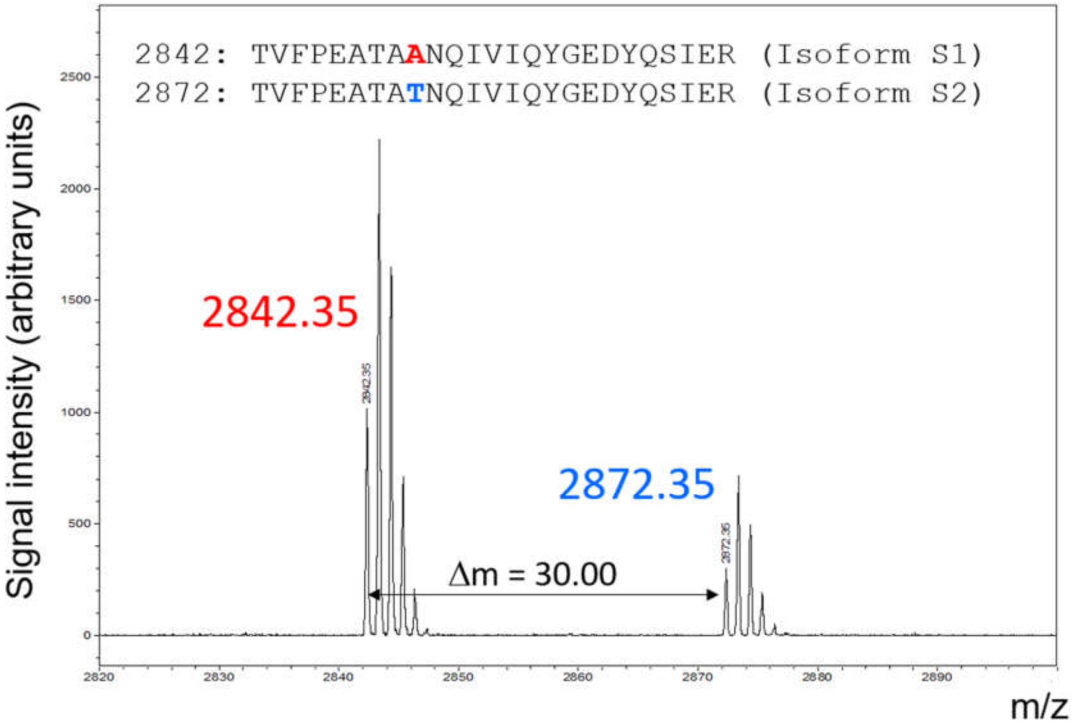

Figure S5. Section of the MS1 spectrum of a tryptic digest of sapovaccarin highlighting the tryptic peptides pos. 102–126 of the isoforms S1 and S2.

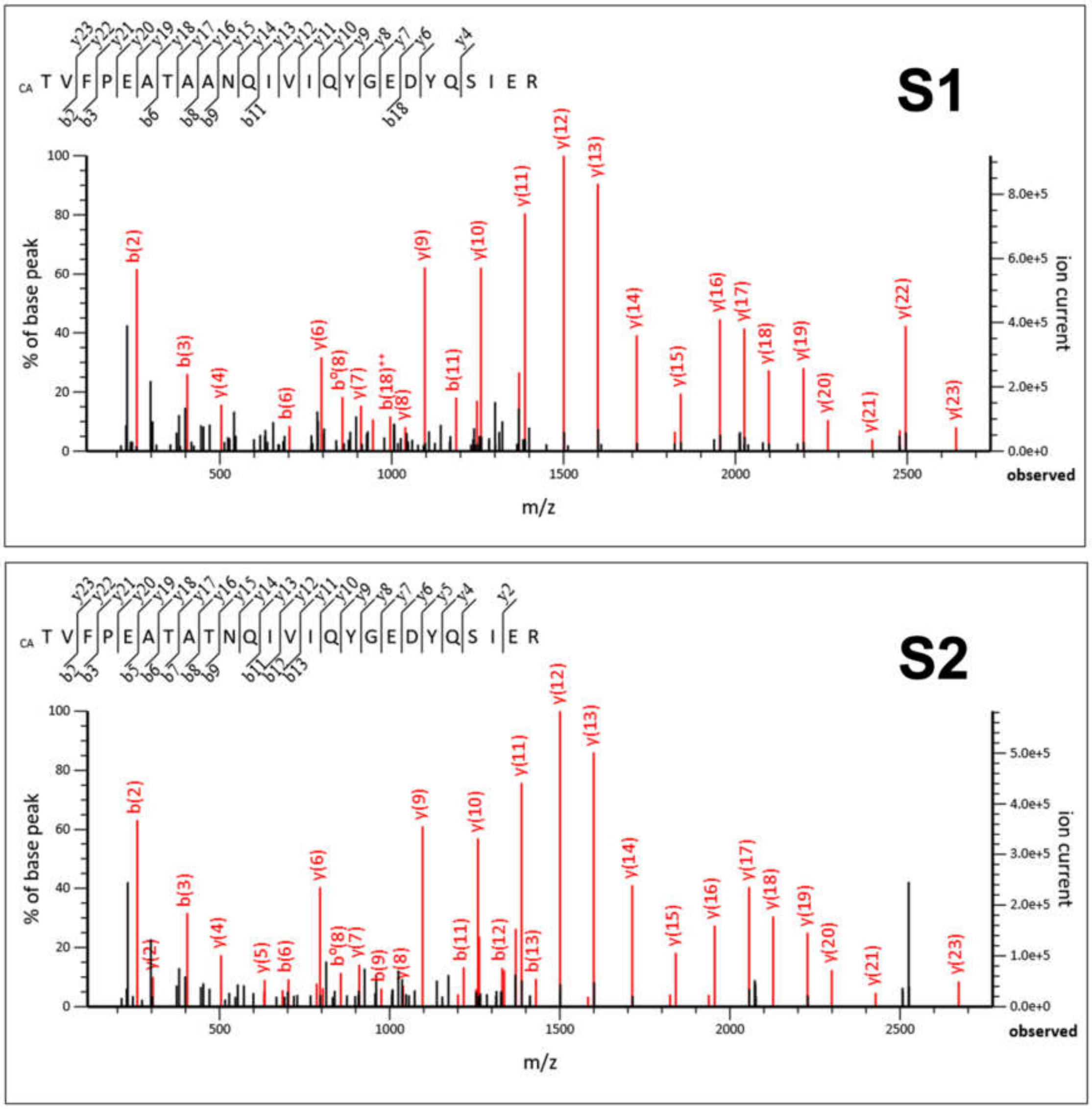

Figure S6. MS/MS spectra of the tryptic peptide pos. 102–126 of the sapovaccarin isoforms S1 (top) and S2 (bottom). Spectra were analyzed using the Mascot MS/MS search software and the corresponding peptide sequences and the matched b- and y-ions are indicated.

S1 (110A)

M+H(mono)=2842.39

b				y
---	1	T	25	---
201.1234	2	V	24	2741.3417
348.1918	3	F	23	2642.2733
445.2445	4	P	22	2495.2049
574.2871	5	E	21	2398.1521
645.3243	6	A	20	2269.1095
746.3719	7	T	19	2198.0724
817.4090	8	A	18	2097.0247
888.4462	9	A	17	2025.9876
1002.4891	10	N	16	1954.9505
1130.5477	11	Q	15	1840.9076
1243.6317	12	I	14	1712.8490
1342.7001	13	V	13	1599.7649
1455.7842	14	I	12	1500.6965
1583.8428	15	Q	11	1387.6124
1746.9061	16	Y	10	1259.5539
1803.9276	17	G	9	1096.4905
1932.9702	18	E	8	1039.4691
2047.9971	19	D	7	910.4265
2211.0604	20	Y	6	795.3995
2339.1190	21	Q	5	632.3362
2426.1510	22	S	4	504.2776
2539.2351	23	I	3	417.2456
2668.2777	24	E	2	304.1615
---	25	R	1	175.1190

S2 (110T)

M+H(mono)=2872.40

b				y
---	1	T	25	---
201.1234	2	V	24	2771.3523
348.1918	3	F	23	2672.2839
445.2445	4	P	22	2525.2154
574.2871	5	E	21	2428.1627
645.3243	6	A	20	2299.1201
746.3719	7	T	19	2228.0830
817.4090	8	A	18	2127.0353
918.4567	9	T	17	2055.9982
1032.4997	10	N	16	1954.9505
1160.5582	11	Q	15	1840.9076
1273.6423	12	I	14	1712.8490
1372.7107	13	V	13	1599.7649
1485.7948	14	I	12	1500.6965
1613.8533	15	Q	11	1387.6124
1776.9167	16	Y	10	1259.5539
1833.9381	17	G	9	1096.4905
1962.9807	18	E	8	1039.4691
2078.0077	19	D	7	910.4265
2241.0710	20	Y	6	795.3995
2369.1296	21	Q	5	632.3362
2456.1616	22	S	4	504.2776
2569.2457	23	I	3	417.2456
2698.2883	24	E	2	304.1615
---	25	R	1	175.1190

Figure S7. Theoretical b- and y-ion series of the tryptic peptide pos. 102–126 of the sapovaccarin isoforms S1 (left) and S2 (right). B-ions differ by 30 Da starting from b9 and y-ions starting from y17.

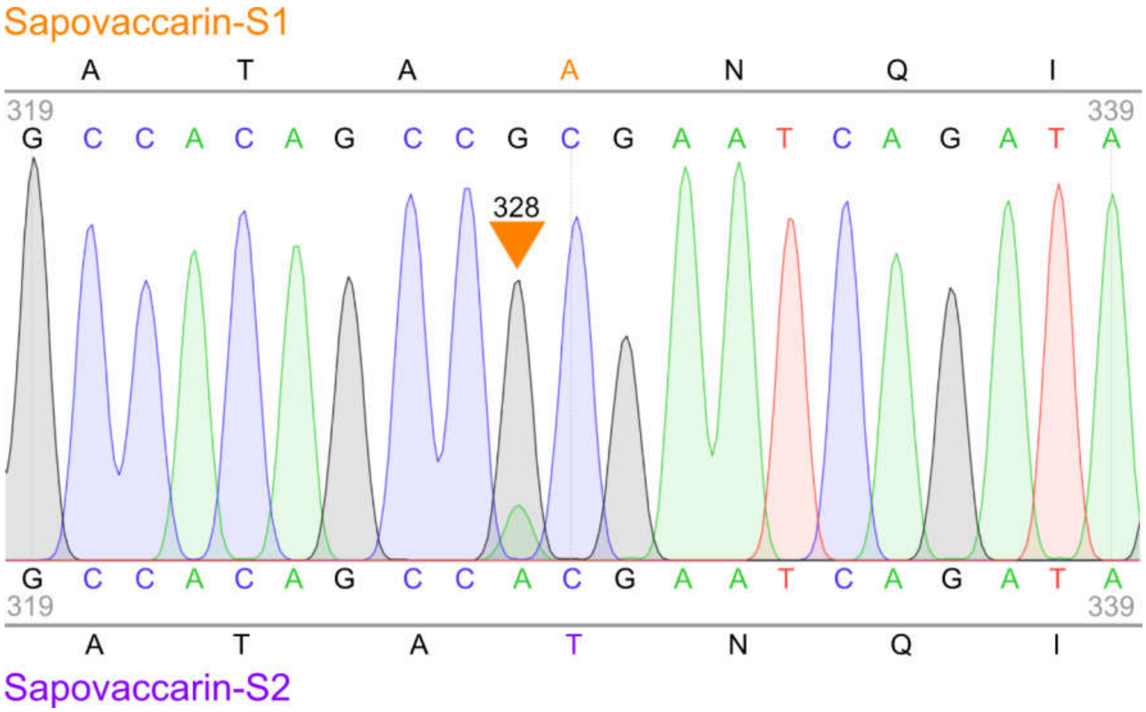

Figure S8. Sequence chromatogram of nucleotides encoding for amino acid positions 107 to 113. At nucleotide position 328 the guanine peak overlays an adenine peak. The codon change from GCG (sapovaccarin-S1) to ACG (sapovaccarin-S2) implicates an amino acid substitution at position 110 from alanine to threonine. Consistent with the results of the trypsin digest and the Orbitrap-based intact protein measurement, the guanine peak is more intense than the adenine peak.
